# Genome-Wide Association Study Identifies Loci and a Candidate Gene Regulating Tomato Seed Germination

**DOI:** 10.3390/ijms27219478

**Published:** 2026-10-23

**Authors:** Chenxu Liu, Yihang Zhu, Jiashun Miao, Hongjian Wan, Zhuping Yao, Meiying Ruan, Qingjing Ye, Zhimiao Li, Guozhi Zhou, Yuan Cheng, Xiaobin Zhang, Rongqing Wang

**Affiliations:** 1 State Key Laboratory for Quality and Safety of Agro-Products, Institute of Vegetables, Zhejiang Academy of Agricultural Sciences, Hangzhou 310021, China; 2 Institute of Digital Agriculture, Zhejiang Academy of Agricultural Sciences, Hangzhou 310021, China; 3 Xianghu Laboratory, Hangzhou 311200, China

**Keywords:** seed germination, genome-wide association study, *SlEXPA10*, tomato

## Abstract

Seed germination is the first critical transition in the plant life cycle, strongly influencing seedling establishment, crop uniformity, and subsequent productivity. However, the genetic basis of seed germination in tomato remains incompletely understood. Here, we performed a genome-wide association study (GWAS) using 319 diverse tomato accessions to identify new loci and candidate genes associated with seed germination. Five significant variants were detected across the genome. Among them, SNP_59508294 on chromosome 3 was consistently associated with the seed germination index (SGI), and accessions carrying the GG haplotype exhibited a significantly higher SGI than those carrying the TT haplotype. Seven *α-expansin* (*EXPA*) genes were located within or near the associated interval. Integration of transcriptomic data from contrasting accessions and expression profiles across tomato tissues showed *SlEXPA10* to be the most plausible candidate gene. The InDels in the *SlEXPA10* promoter were highly associated with its low expression level in low SGI varieties. CRISPR/Cas9-mediated disruption of *SlEXPA10* significantly reduced the rate and speed of seed germination relative to the wild type. Collectively, these results identify SlEXPA10 as a positive regulator of tomato seed germination and provide a candidate locus for improving seed vigor and emergence uniformity.

## 1. Introduction

Seed germination is the first developmental transition in the plant life cycle, representing a decisive stage during which a seedling has only one opportunity to establish successfully [1]. Morphologically, germination is complete when the seed coverings are ruptured, and the radicle protrudes [2]. Physiologically, this process involves the reactivation of metabolism, the mobilization of stored reserves, and the induction of hydrolytic enzymes that supply energy and structural components for early seedling growth [3]. Rapid and uniform germination therefore contribute directly to stand establishment, vegetative growth, and yield formation [3]. Seed germination is controlled by complex interactions among genetic factors, environmental cues, and phytohormonal signaling pathways [4,5]. Identifying the loci and genes underlying variation in germination is thus important for improving germplasm with poor seed vigor or uneven emergence.

Over the past several decades, numerous quantitative genetic loci (QTL) associated with seed dormancy and germination have been identified [6,7]. In *Arabidopsis*, a series of *DELAY OF GERMINATION* (*DOG*) loci have been characterized. *DOG1* is a major regulator of seed dormancy and acts through mechanisms closely associated with the abscisic acid (ABA) signal transduction pathway [8]. *REDUCED DORMANCY5* (*RDO5*), also known as *DOG18*, encodes a protein phosphatase 2C and regulates dormancy partly by affecting the transcription of ARABIDOPSIS PUMILIO9 and PUMILIO11 [9]. Germination-related loci have also been identified in cereal crops. In rice, *Seed Dormancy 6* (*SD6*) and *INDUCER OF C-REPEAT BINDING FACTOR EXPRESSION 2* (*ICE2*) antagonistically regulate seed dormancy and germination by modulating ABA metabolism [10]. In addition, functional genes associated with cell wall softening and cell expansion are also reported to regulate seed germination process, including *xyloglucan endotransglucosylase/hydrolase* (*XTH*), *endo-β-mannanase* (*MAN*), and *expansin* (*EXP*) [11,12,13].

*Expansins* are a family of cell wall-loosening proteins that function by disrupting the non-covalent hydrogen bonds between cellulose microfibrils and matrix polysaccharides [14,15]. The expansin family is divided into four subgroups, namely α-expansin (EXPA), β-expansin (EXPB), expansin-like α (EXLA), and expansin-like β (EXLB) [16]. EXPA and EXPB are primarily involved in cell wall loosening and cell expansion [17]. The functions of EXLA and EXLB remain less explored, with a few studies implicating them in stress tolerance [18]. EXPAs and EXPBs have been reported to participate in a wide range of developmental processes, including seed germination, root hair initiation and elongation, leaf development, stem elongation, pollen tube growth, and fruit ripening [17,19,20]. For instance, *SlExp1* is one member of the EXPA gene family in tomato, which positively regulates fruit softening [21]. LeEXP4 may regulate seed germination by contributing to cell wall disassembly associated with endosperm cap weakening [22]. In addition, *LeEXP8* and *LeEXP10* are expressed during tomato seed germination [23]. However, the functions of three *EXPs* in regulating tomato seed germination need more evidence. In *Stylosanthes*, *SgEXPB1* promotes phosphorus absorption and root growth [24]. In addition, EXPAs/EXPBs are also involved in responses to both abiotic and biotic stresses [25,26]. *HvEXPB7* positively regulates root hair growth and potassium uptake under drought stress [26]. In tobacco, overexpressing *NbEXPA1* promotes the replication and movement of *Turnip mosaic virus* (TuMV), thereby facilitating virus infection [25].

Advances in genome sequencing and bioinformatics have enabled increasingly powerful dissection of the genetic architecture of complex traits [27]. Genome-wide association studies (GWASs) evaluate associations between phenotypic variation and genome-wide polymorphisms in natural populations or breeding panels [28]. Single nucleotide polymorphisms (SNPs) and small insertions/deletions (InDels), typically generated through high-throughput resequencing, are the most commonly used marker types [29]. By identifying variants that are significantly associated with target traits, GWAS can facilitate the localization of candidate genes and potentially functional alleles [30]. This strategy has been widely applied to seed germination and related traits in crops [4,31,32]. For example, combined GWAS and transcriptome analyses implicated MADS26 in maize seed germination, with its expression during germination being responsive to ethylene [31]. In super-sweet corn, GWAS identified loci and candidate genes associated with seedling emergence [32]. In soybean, GmAOC4 was shown to regulate germination by affecting jasmonic acid biosynthesis [4].

Tomato (*Solanum lycopersicum*) is an economically important vegetable plant cultivated worldwide, due to its distinctive flavor [33]. GWAS has been extensively used in elucidating tomato agronomic traits and fruit quality, including fruit weight, flowering time, soluble sugar, organic acid and volatile organic compounds [33,34,35,36]. In contrast, the genetic mechanisms underlying natural variation in tomato seed germination remain poorly characterized. This study was designed to identify genetic loci associated with tomato seed germination using GWAS and to validate the functional relevance of the prioritized candidate gene. We hypothesized that seed germination is controlled by discrete genetic loci that can be detected through GWAS analysis, and that resolving such loci would provide candidate targets for improving tomato seed vigor.

## 2. Results

### 2.1. The Population Structure, Principal Component and Phylogenetic Analysis of 319 Tomato Accessions

Based on the results of whole genome resequencing, the variation analysis identified 12392992 high-quality SNPs in 319 tomato accessions. The phylogenetic tree revealed a certain clustering trend among different tomato groups. The number of accessions belong to *S. pimpinellifolium*, *S. lycopersicum* var. *cerasiforme* and *S. lycopersicum* was 9, 26 and 284, respectively (Table S1). One wild tomato, *S. lycopersicoides*, was added into the phylogenetic tree analysis. As shown in Figure 1A,B, wild relative species materials, such as *S. lycopersicoides* and *S. pimpinellifolium*, were primarily distributed on the outer branches or earlier divergent branches of the phylogenetic tree, corresponding to the peripheral positions in the PCA plot. Cultivated tomatoes (*S. lycopersicum*) formed the main component of the samples and exhibited multiple internal subgroups, occupying the central and most extended regions of the plot (Figure 1A,B). The two clusters within *S. lycopersicum* likely reflect the well-documented genetic differentiation between modern processing/fresh-market varieties and heirloom accessions, or between different breeding lineages. The cherry tomato type (*S. lycopersicum* var. *cerasiforme*) was distributed between the branches related to wild species and cultivated tomatoes or nested within some cultivated tomato branches, indicating its genetic background possesses transitional and complex characteristics (Figure 1A). Admixture analysis further revealed genetic composition differences among the materials. At K = 3, most cultivated tomatoes were predominantly composed of a single ancestral component, while materials at both ends of the phylogenetic tree and specific branches exhibited enriched ancestral components. As K increased to K = 5, the original large population was further subdivided, with multiple genetic subgroups emerging within cultivated tomatoes. Some materials displayed more pronounced mixed ancestral components. At K = 9, the structure became even more detailed, showing more subgroups within cultivated tomatoes, more accessions with mixed ancestry, and clearer separation between wild and cultivated species. Overall, the population structure differentiation showed good consistency with the major branches of the phylogenetic tree, but no completely independent genetic units formed between species or types, suggesting potential shared ancestors, historical gene exchange, or genetic component introgression during breeding among different tomato groups.

### 2.2. GWAS Identified Major Loci Associated with Tomato Seed Germination

To investigate the significant quantitative genetic loci (QTL) of tomato seed germination, we identified the seed germination rate of 319 tomato accessions over 7 days. To comprehensively evaluate the seed germination rate and seed germination potential, we calculated the seed germination index (SGI) for further GWAS analysis. The SGI of 319 tomato accessions showed nearly normal distributions (Figure 1C). Total 12392992 SNPs from 319 tomato accessions were filtered and used for GWAS analysis. The genomic inflation factors were λ = 3.1498 for GLM, λ = 1.281 for MLM, and λ = 0.9571 for FarmCPU (Figure S1). The GLM showed substantial inflation (λ = 3.1498), indicating that its association signals were likely confounded by false positives. The MLM reduced inflation considerably (λ = 1.281), but no SNP exceeded the genome-wide significance threshold (Figure 2), suggesting that the stringent correction for relatedness may have removed both false and true associations, or that the signal is diluted under this model. In contrast, FarmCPU showed minimal inflation (λ = 0.9571) and identified SNPs exceeding the significance threshold (Figure 2C). The manhattan plot of the FarmCPU model showed five significant SNPs associated with SGI, using threshold of 8.39 (Figure 2). Five significant SNPs were distributed in chromosomes 1, 3, 4, 10 and 12, respectively. The −log_10_(*P*) value of five SNPs ranged from 8.94 to 13.61. SNP_65092668 in chromosome 12 showed the highest −log_10_(*P*) value, and SNP_10222208 exhibited the lowest −log_10_(*P*) value (Table 1). The seed germination index of all significant loci was evaluated and three loci showed significant difference between two haplotypes, including SNP_59508294, SNP_58461279 and SNP_50270468 (Figure 3A and Figure S2). Meanwhile, we noticed that SNPs in chromosome 3 exhibited a significant −log_10_(*P*) value in both the GLM and FarmCPU model. Hence, we hypothesize that the genomic interval near SNP_59508294 in chromosome 3 is highly associated with tomato seed germination.

Moreover, we also performed GWAS analysis with principal component covariates. We found five significant loci, and a significant locus in chromosome 3 was in the same region as SNP_59508294 (Figures S3 and S4). The other four significant loci identified contained a considerable number of heterozygous haplotype or reference haplotype (Table S2). Although principal component covariates were included to control for population stratification, the resulting loci showed no improvement in reliability, as the additional loci were predominantly associated with heterozygous or reference haplotypes rather than clear allelic effects. Therefore, the original GWAS results were retained for candidate gene prediction.

### 2.3. Haplotype Analysis and Candidate Gene Prediction

According to the results of the GWAS, we selected a genomic interval in chromosome 3 for haplotype analysis and candidate gene prediction. As shown in Figure 3B, physical position of 59460000 to 59558000 which near SNP_59508294 was selected for haplotype analysis. The average seed germination rates of Hap I and Hap II on SNP_59508294 were calculated. The results showed that the seed germination index of Hap II (GG) was significantly higher that of Hap I (TT). The SNP_59508294 was located 1265 bp upstream of *EXPA23*. To further identify candidate genes, we listed all genes within 50 kb upstream and downstream of SNP_59508294 (Table S3). We identified seven *α-Expansin* genes (*SlEXPA*) in this interval which were previously identified as responsible for cell wall expansion and reported to participate in tomato seed germination [23]. Based on prior research, these seven *Expansin* genes were named: *SlEXPA7*, *SlEXPA10*, *SlEXPA19*, *SlEXPA20*, *SlEXPA21*, *SlEXPA22*, *SlEXPA23*. Except for *SlEXPA10*, the other six genes were clustered together in the 16.1 kb region. *SlEXPA10* was also located in chromosome 3 near the other six genes (399 kb). Previous studies have shown that the *Expansin* gene family is associated with cell expansion, cellulose degradation and seed germination in multiple plants [3,16,37]. Hence, we predict that these seven genes may be candidate genes for the regulation of tomato seed germination.

### 2.4. SlEXPA10 Is a Potential Candidate Gene

To further identify the functional gene in regulating tomato seed germination, we analyzed the expression pattern of *SlEXPA* genes in extreme materials and different tomato tissues. Firstly, we performed RNA-seq analysis of two extreme materials under seed germination. C4 was a high SGI material with strong germination potential and its haplotype of SNP_59508294 was GG, C31 was a low SGI material with weak germination potential and its haplotype of SNP_59508294 was TT. RNA-seq data identified 5918 differential expressed genes between C31 and C4, of which 2027 genes were upregulated and 3891 genes were down-regulated (Figure 4A). The transcript levels of *SlEXPA7*, *SlEXPA19*, *SlEXPA20*, *SlEXPA21*, *SlEXPA22* and *SlEXPA23* were extremely low. In contrast, *SlEXPA10* exhibited markedly higher expression, with its expression level in C4 being approximately three times that observed in C31 (Figure 4B). We also analyzed the expression level of different tomato tissues and organs based on previous research [38]. The heatmap showed extremely low expression levels of the other six *expansin* genes in all tomato tissues and organs (Figure 4C). Only *SlEXPA10* showed a high expression level in seed. The time course of the *SlEXPA10* expression level during seed germination was also determined. As shown in Figure 4D, the transcript abundance of *SlEXPA10* reached a peak at 48 h after seed imbibition, and then gradually reduced. However, more genetic evidence is required for this inference.

### 2.5. SlEXPA10 Positively Regulates Tomato Seed Germination

To further verify the function of *SlEXPA10* in seed germination, we constructed an *expa10* knockout line using CRISPR-Cas9 technology. The mutant site of *SlEXPA10* was detected using PCR analysis and one base pair (T) was inserted into the first exon of *SlEXPA10*, which resulted in early translation termination (Figure 5A). The phenotype and seed germination rate of *expa10* and WT materials were investigated for 7 days after seed imbibition (Figure 5B,C). The results demonstrated that, compared with the *SlEXPA10*-knockout line, WT seeds exhibited faster germination and a higher germination rate, directly confirming that *SlEXPA10* positively regulates tomato seed germination.

### 2.6. InDels in the SlEXPA10 Promoter May Influence Its Gene Expression Level

To investigate how the gene expression level of *SlEXPA10* is influenced in different tomato accessions, the variation in the promoter of *SlEXPA10* was investigated and this promoter contained two insertion/deletion (InDel) variations in complete linkage (Figure 6A). The seed germination index of the insertion haplotype (C31) was significantly lower than the deletion haplotype (C4) (Figure 6B). Dual-luciferase reporter assays revealed that the promoter activity of the insertion haplotype, as reflected by the LUC/REN ratio, was significantly lower than that of the deletion haplotype (Figure 6C). Given that promoter activity is closely associated with gene expression levels, we propose that the insertion in the *SlEXPA10* promoter is associated with its reduced expression in tomato varieties with low SGI, thereby contributing to the observed phenotypic variation in germination. Moreover, we found that the SNP_59508294 identified by GWAS is not genetically linked to InDels in *SlEXPA10* promoter. These results indicated that the InDel in *SlEXPA10* may influence its gene expression during seed germination process.

## 3. Discussion

Uniform and rapid seed germination determinates seedling development and crop production [1]. Previous studies have elucidated the genetic mechanism of seed germination in crops including rice, maize and wheat [5,6,31,39]. However, research in tomato has focused more on agronomic traits or fruit quality [33,35,40]. Studies on seed germination is relatively rare, especially using traditional genetic mapping. We performed a comprehensive GWAS analysis on a diverse panel of 319 tomato accessions to determine the genetic architecture underlying seed germination. Our findings identified five significant loci associated with the seed germination index (SGI), with the locus on chromosome 3 (SNP_59508294) exhibiting robust signals across multiple models. Through integrated haplotype and transcriptomic analyses, we pinpointed *SlEXPA10*, a member of the *expansin* gene family, as a strong candidate gene. Crucially, CRISPR-Cas9 mediated knockout of *SlEXPA10* resulted in significantly reduced germination, providing direct genetic evidence that *SlEXPA10* positively regulates tomato seed germination. Furthermore, the promoter analysis indicated that the InDel in the *SlEXPA10* promoter region may lead to its difference in expression in extreme materials. This study identified a new gene mediating seed germination in tomato, which provides a potential target for genetic improvement.

GWAS has emerged as a powerful tool for dissecting the genetic basis of the natural variation in seed germination, due to its high resolution and high efficiency [29,31,41]. In our research, the comparison of GWAS models revealed marked differences in genomic inflation and detection power. The GLM showed a high inflation factor (λ = 3.1498), consistent with inadequate control of the population structure and kinship, and its associations should therefore be interpreted with caution. The MLM reduced inflation to λ = 1.281, but no SNP reached genome-wide significance. This may reflect over correction and the model may be overly conservative, thus losing the power to detect true associations. Alternatively, the SGI signal may be modest relative to the stringent threshold applied. FarmCPU, which iteratively uses fixed-effect models and a kinship-based variance component, yielded λ = 0.9571, indicating minimal inflation. We identified five significant loci related to tomato seed germination trait using this model. The genomic inflation factor of FarmCPU reflected its reliability compared with other models in analyzing the seed germination index (Figure S1). Among the identified loci, SNP_59508294 on chromosome 3, SNP_58461279 on chromosome 4 and SNP_50270468 on chromosome 10 were the dominant loci (Figure S2). These three loci were all on the promoter region of candidate genes, including *Solyc03g115340*, *Solyc04g074510* and *Solyc10g050960*. *Solyc03g115340* is annotated as an *expansin* gene, reported to regulate the seed germination process [3,12]. *Solyc04g074510*, was annotated as a 14-3-3 protein, and *Solyc10g050960* was annotated as an ethylene-responsive transcription factor. In rice, the 14-3-3 protein OsGF14h boosts seed germination under anaerobic conditions [42]. In *Arabidopsis*, the ERF1 transcription factor promotes seed germination through repressing the jasmonic acid signaling pathway [43]. Given the well-established role of *expansins* in cell wall loosening during germination, we selected the chr3 locus for further analysis.

Recently, several genes have been identified to influence tomato seed germination, such as *SlLEA2*, *MAN2*, *MAN3*, *EXP4*, *EXP6* and *XTH23* [3,44,45]. The function of these genes is concentrated on endosperm weakening and cell wall relaxation and expansion [20]. In our study, the *expansin* gene cluster was identified using GWAS analysis. Expansins are known to mediate cell wall loosening by disrupting hydrogen bonds between cellulose and hemicellulose, a process essential for radicle protrusion [20,37]. Our results align with these previous biochemical and physiological observations, extending them to tomato. Unlike previous studies that primarily focused on model plants such as *Arabidopsis* (e.g., *DOG* loci) or cereals (e.g., *SD6* in rice), our GWAS approach successfully translated these conserved mechanisms to a horticultural crop [8,10].

The transcriptomic analysis provided a compelling filter for prioritizing candidate genes among the seven *SlEXPA* genes clustered on chromosome 3. While all seven genes were initially considered candidates based on their physical distance, the expression profiling revealed a significant distinction. The six *SlEXPA* genes (*SlEXPA7*, *SlEXPA19*-*SlEXPA23*) exhibited extremely low or undetectable expression levels in both germinating seeds and across various tomato tissues. Noticeably, the latest research has disclosed that six clustered *expansins* are expressed on flower pedicels, controlling the tomato pedicel length [40]. In contrast, *SlEXPA10* showed a germination-specific expression pattern, with its transcript abundance peaking at 48 h post-imbibition which is a critical time point coinciding with radicle emergence in tomato. These expression dynamics strongly suggest that *SlEXPA10* is the principal functional contributor in this gene cluster. The differential expression analysis between the high-SGI (C4) and low-SGI (C31) extreme materials further verified this hypothesis. A previous study showed that *SlEXPA10* is highly expressed at the early stage of seed development and its expression level peaked on 12 h after seed imbibition [23]. This result is inconsistent with our study, and the difference is attributed to the fact that the detection technique of the gene expression level in this previous research was less accurate than qRT-PCR, which is broadly used in current biological research. Two studies revealed that *SlEXPA10* is specifically expressed in the tomato seed germination process, which confirms its function in seed germination.

The promoter of *SlEXPA10* in two extreme genotypes was analyzed, with two InDel polymorphisms identified. The seed germination index analysis and promoter activity assay both indicated that the insertion haplotype induced a low expression of *SlEXPA10*. InDels in a promoter affecting downstream gene expression have been documented in several species, including tomato, apple, and *Prunus mume* [46,47,48]. For instance, a 3 bp insertion in the promoter of *SlALMT9* resulted in its high expression and a high malate content in tomato fruit [46]. A 58 bp insertion in the *MdNAC18.1* promoter activated its expression and conferred early ripening in apple [47]. In contrast to these activation cases, the insertion in the *SlEXPA10* promoter identified in our study appears to repress transcription. Similarly, in *Prunus mume*, the insertion of AG into the *PmARF9* promoter correlated with reduced expression [48]. Nevertheless, the specific InDel responsible for the repression of *SlEXPA10* and the transcription factors that potentially bind to this region remain to be determined.

One notable observation is that the difference in promoter activity between the two haplotypes in the LUC/REN assay (Figure 6C) was substantially larger than the difference in transcript abundance detected via RNA-seq. Several factors may account for this discrepancy. First, the LUC/REN assay is a heterologous transient expression system in tobacco leaves, in which the tomato promoter is tested outside its native chromatin, trans-acting factor, and tissue context. Tobacco leaves may differ from tomato seeds in the abundance or activity of the transcription factors that bind these promoter variants, potentially amplifying haplotype-dependent differences that are buffered in the native seed context. Second, the two assays measure different quantities: the LUC/REN ratio reflects promoter-driven transcriptional activity in a transient, episomal context, whereas RNA-seq measures steady-state mRNA abundance in developing seeds, which integrates transcriptional, post-transcriptional, and degradation effects, as well as cell-type heterogeneity within the seed. Third, the transient assay typically involves the strong overexpression of the reporter construct and may therefore exaggerate differences that are attenuated under endogenous regulatory conditions. Therefore, the LUC/REN result should be interpreted as evidence that the two promoter haplotypes differ in intrinsic transcriptional activity, rather than as a direct quantitative predictor of the expression difference in plants.

Genetic evidence is indispensable for verifying the function of *SlEXPA10* in seed germination. The *expa10* knockout line generated using the CRISPR technique displayed a significant reduction in both the germination rate and speed compared to the wild type, demonstrating that *SlEXPA10* is a positive regulator of tomato seed germination. Expansin proteins are responsible for cell wall loosening which is essential for the cell elongation process [20]. During germination, the weakening of the endosperm cap is a prerequisite for radicle protrusion in many dicot species, including tomato [3]. It is plausible that the SlEXPA10 protein facilitates this weakening by promoting cell wall extensibility, thereby reducing the mechanical resistance that the emerging radicle must overcome [2]. According to previous research, the expression of *expansins* is independent of or integrated with phytohormone signaling pathways or transcription factors, which are known to orchestrate germination [19]. In tomato, LBD40 inhibits seed germination through suppressing *EXP6* and *XTH23* expression levels [3]. In *Arabidopsis*, exogenous GA treatment increases *EXP2* gene expression during the seed germination process [12].

However, several limitations of this study should also be acknowledged. Firstly, we used SGI for GWAS analysis and the seed germination rate during days 1–2 was not included, which may have decreased the precision in the GWAS analysis. Secondly, while our GWAS panel encompassed three *Solanum* species (*S. lycopersicum*, *S. lycopersicum* var. *cerasiforme*, and *S. pimpinellifolium*), the functional validation was performed exclusively in the ‘Ailsa Craig’ background. The effect of *SlEXPA10* on germination in other genetic backgrounds, particularly those with different haplotypes, deserves further investigation. Thirdly, future research should focus on unraveling the upstream regulatory network controlling *SlEXPA10* expression, including transcription factors and epigenetic modifications, as well as the potential interplay between SlEXPA10 and phytohormone signaling pathways during germination [40]. How the transcription factor binds the InDel region and represses *SlEXPA10* expression level during the seed germination process needs to be clarified. In addition, SNP_59508294 on chromosome 3 was identified in our GWAS analysis and this SNP is not linked to the natural variation in *SlEXPA10*. Nevertheless, the functional link between this SNP and *SlEXPA10* remains to be elucidated, particularly whether this variant exerts long-range regulatory effects on gene expression. Finally, research associated with the seed germination process in crops is more concentrated on abiotic stress conditions, including salinity, drought, or temperature stresses, rather than optimal conditions. It remains to be explored whether *SlEXPA10* also contributes to germination under stress conditions, which would be of great agronomic significance.

## 4. Materials and Methods

### 4.1. Plant Material and DNA Extraction

All 319 tomato accessions, comprising *Solanum lycopersicum*, *Solanum lycopersicum* var. *cerasiforme,* and *Solanum pimpinellifolium,* were collected from United States Department of Agricultural (USDA), the Institute of Vegetable of the Zhejiang Academy of Agricultural Sciences and commercial breeding companies. The detailed information of the tomato accessions is listed in Supplementary Table S3. Genomic DNA was extracted from young leaf tissue using a standard cetyltrimethylammonium bromide (CTAB) [49].

### 4.2. Measurement of Seed Germination Index

Seeds from the 319 accessions were imbibed in double-distilled water (ddH_2_O) and incubated in darkness for 7 days. Three biological replicates were performed for each tomato accession, with 100 seeds used per replicate. A seed was considered germinated when radicle protrusion through the seed coverings was visible. The seed germination rate on day i (SGRi) was calculated as SGRi = Gi/N × 100%, where Gi is the cumulative number of germinated seeds on day i after imbibition and N is the total number of seeds tested. The seed germination index (SGI), which integrates the germination percentage and germination speed, was calculated as SGI = SGR3 × 7 + SGR4 × 6 + SGR5 × 5 + SGR6 × 4 + SGR7 × 3.

### 4.3. Whole-Genome Resequencing and Variant Calling

Genomic DNA libraries were sequenced on an Illumina platform by Novogene Co., Ltd. (Beijing, China). After DNA quality assessment, 0.2 μg of genomic DNA per accession was used for library construction with the NEBNext Ultra DNA Library Prep Kit for Illumina (E7370L; New England Biolabs, Ipswich MA, USA), following the manufacturer’s instructions. Raw reads were generated in FASTQ format and subjected to quality control. High-quality reads were aligned to the tomato reference genome SL4.0 using the Burrows-Wheeler Aligner (BWA) [50]. The resulting BAM files were sorted, and duplicate reads were marked or removed using SAMtools v1.20 and Picard tools (https://broadinstitute.github.io/picard/, accessed on 10 July 2024) [51]. Variants were called using the Genome Analysis Toolkit v4.2.1.0 (GATK) [52]. SNPs were retained when the minor allele frequency was ≥0.05 and the missing-genotype rate was ≤0.20.

### 4.4. Population Structure, Phylogenetic Analysis, and Principal Component Analysis

Phylogenetic relationships were inferred from genome-wide SNPs using FastTree v2.2.0, with branch support evaluated using 1000 replicates [53]. The resulting tree was visualized and annotated using the Interactive Tree of Life v7 (iTOL) [54]. The population structure was assessed using ADMIXTURE v1.3.0 [55]. Principal component analysis (PCA) was performed in PLINK v1.9 using the command --pca 20 [56].

### 4.5. Genome-Wide Association Study of Seed Germination Index

GWAS was performed using the general linear model (GLM), mixed linear model (MLM), and fixed and random model circulating probability unification (FarmCPU) implemented in the R package rMVP [28]. A genomic kinship matrix was calculated using rMVP and incorporated into the MLM to account for genetic relatedness. No principal components or other population-structure covariates were included as fixed effects in the GLM, MLM, or FarmCPU analyses. For FarmCPU, confounding due to population structure and relatedness was addressed through its iterative fixed-effect and random-effect modeling framework with pseudo-QTN (Quantitative Trait Nucleotide) selection. The remaining parameters were set according to the rMVP default settings. Haplotype analysis for the candidate interval was performed using Haploview v4.0 [57]. Linkage disequilibrium (LD) blocks were defined using a threshold of *r*^2^ > 0.9.

### 4.6. RNA Isolation, Transcriptome Analysis, and qRT-PCR Analysis

Total RNA of tomato seed was extracted using HiPure Plant RNA Plus Kit (R4150, Magen Biotechnology, Guangzhou, China). The transcriptome sequencing was performed by Annoroad Gene Technology (Beijing) Co., Ltd. In detail, the mRNA was enriched using Oligo (dT) magnetic beads, and then fragmentation buffer was added for mRNA fragmentation. cDNA was synthesized using mRNA as a template, and cDNA was subjected to end repair, base A addition, and sequencing adapter addition. The denatured double stranded cDNA into single stranded DNA at high temperature, added cyclization primers, and formed a single stranded circular library. The library-quality statistics information is listed in Supplementary Table S4. Sequencing was performed on the DNBSEQ-T7 platform. Raw data were filtered and high-quality data were mapped to the tomato reference genome (SL4.0 from ITAG) using HISAT2 v2.2.0 software [58]. Differential expression analysis was performed using DESeq2 v1.52.0 software [59]. For each accession, three biological replicates (independent plants) were used for RNA-seq. DEGs were identified using DESeq2 with an adjusted *p*-value (FDR) threshold of <0.05 and an absolute log_2_ fold-change threshold of ≥1.

For qRT-PCR, the cDNA was generated using Hiscript II qRT Supermix for qPCR reagent (R223, Vazyme, Nanjing, China). qRT-PCR was performed on a CFX96 real-time system (Bio-Rad, Hercules CA, USA). The reaction mixture included 0.4 μL forward primer, 0.4 μL reverse primers, 2 μL cDNA template, 7.2 μL ddH_2_O, and 10 μL SupRealQ Purple Universal SYBR qPCR Master Mix reagent (Q412, Vazyme, Nanjing, China). The primer used for qRT-PCR is listed in Supplementary Table S5.

### 4.7. Generation of CRISPR-Cas9 Mediated Mutant Plant

The mutant line of *SlEXPA10* gene was generated using the CRISPR/Cas9 technique on the wild type tomato variety ‘Ailsa Craig’. The CRISPR/Cas9 vector was constructed as previously described [60]. In detail, the target sequence was designed using the CRIPSR-P website (http://crispr.hzau.edu.cn/CRISPR2/, accessed on 16 June 2025). The target sequence of *SlEXPA10* was inserted into the pHEE401 vector and tomato genetic transformation was performed as previously described. The mutant site of *SlEXPA10* was cloned via PCR and sequence alignment was conducted using DNAMAN v9 software. The Cas9-free mutant line was used for further experiments.

### 4.8. Dual-Luciferase Assay

The two haplotypes promoter of *SlEXPA10* (−600 to −1 bp) were amplified using C4 and C31 genomic DNA as templates. The fragments were then cloned to the pGreenII 0800-LUC vector and transformed into *A. tumefaciens* strain GV3101. The *A. tumefaciens* strains were then adjusted to OD600 = 0.75 using an infection solution (10 mM MES, 10 mM MgCl_2_ pH 5.6, 150 µM acetosyringone) and injected into tobacco leaves (*N. benthamiana*). At 48 to 72 h after infiltration, the firefly luciferase activity and renilla luciferase activity were detected using a Dual Luciferase Reporter Assay Kit (Vazyme DL101, Nanjing, China). The promoter activity was calculated according to the ratio of LUC/REN. Each sample contained four biological replicates and every biological replicate had three technical replicates.

### 4.9. Statistical Analysis

Data analysis was performed using R software (Version 4.2.0; https://www.r-project.org/) and the results are reported as the mean ± standard deviation (SD). Comparisons between multiple groups were assessed using one-way ANOVA or two-way ANOVA as specified in the figure legends. The detailed information of the statistical analysis is described in the figure legends.

## 5. Conclusions

In this study, we integrated GWAS, transcriptomics, and reverse genetics and identified *SlEXPA10* as a key positive regulator of tomato seed germination. Our findings not only address the knowledge gap regarding the genetic basis of seed germination in tomato but also provide a valuable genetic resource for breeding programs aimed at improving seed vigor and uniform emergence in tomato cultivation.

## Figures and Tables

**Figure 1:**
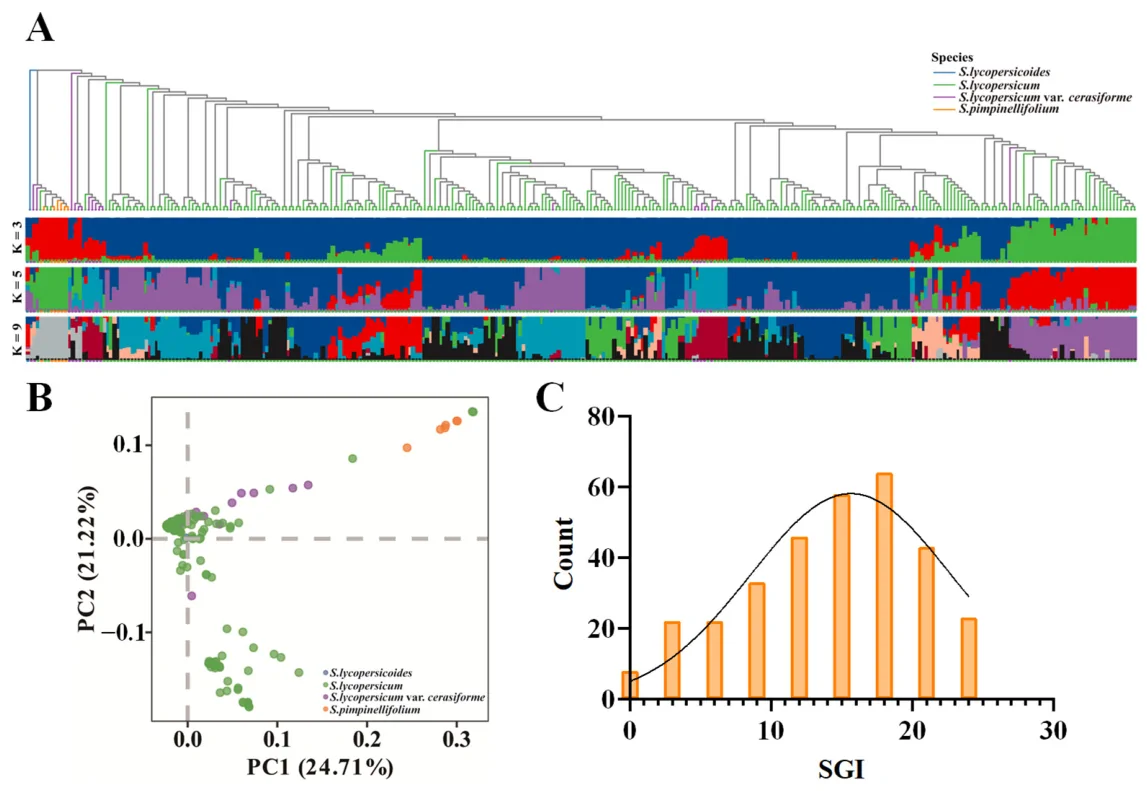
Population structure, phylogenetic relationship and seed germination index histograms of 319 tomato accessions. (**A**) Model-based clustering analysis of 319 tomato accessions based on different numbers of ancestry kinship (*K* = 3, 5, 9). (**B**) Principal component analysis (PCA) of 319 tomato accessions. (**C**) The frequence distribution of seed germination index.

**Figure 2:**
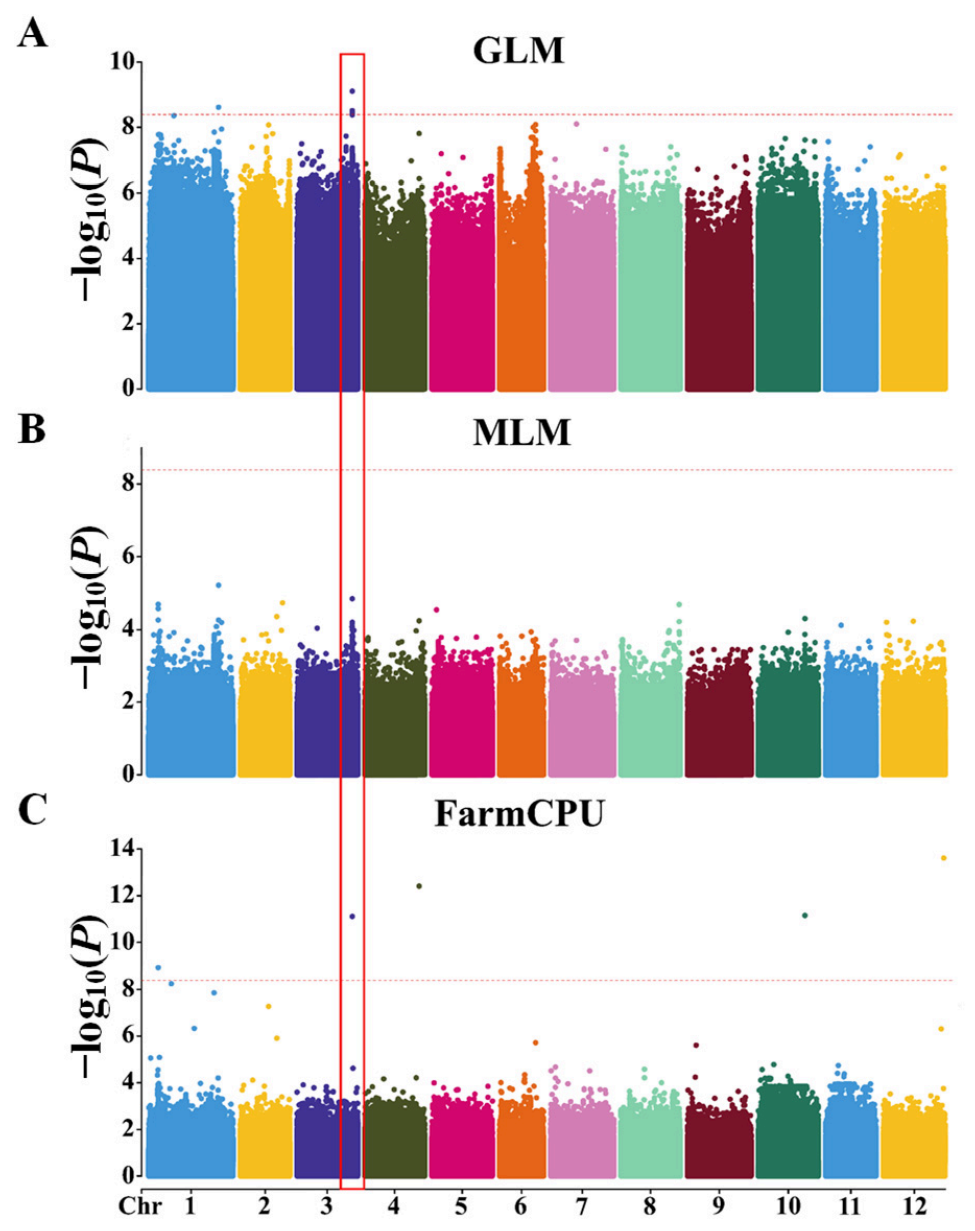
The genome wide association and genetic effect analysis of the tomato seed germination index. (**A**) GWAS analysis of tomato seed germination using the General Linear Model (GLM). (**B**) GWAS analysis of tomato seed germination using the Mixed Linear Model (MLM). (**C**) GWAS analysis of tomato seed germination using the Fixed and random model Circulating Probability Unification (FarmCPU) model. The dashed horizontal line represents the threshold of GWAS analysis (−log_10_(*P*) = 8.39). The red rectangle represents the region of SNP_59508294.

**Figure 3:**
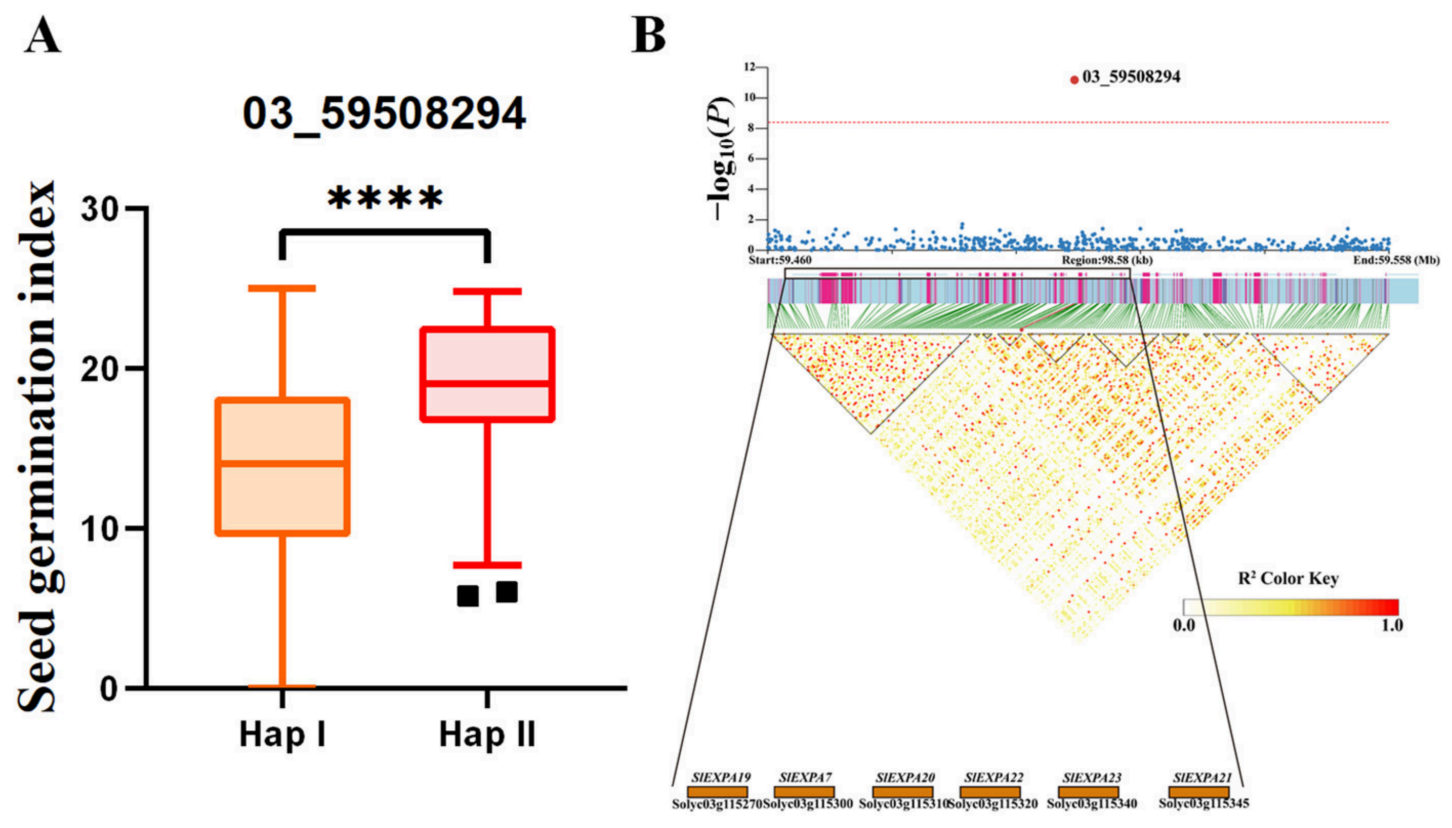
Haplotype analysis and candidate gene prediction. (**A**) The seed germination index of two haplotypes of SNP_59508294. Hap I is TT haplotype and HapII is GG haplotype. Significant difference was calculated using one-way ANOVA (**** *p* < 0.0001). (**B**) Linkage disequilibrium plot for SNPs in the region of 59.460 to 59.558 Mb. SNP_59508294 is labeled with a red blot, and six *SlEXPA* genes in this region were marked out. Each cell shows the pairwise R^2^ between two SNPs, and the color scale indicates the LD strength, from white (R^2^ = 0) to red (R^2^ = 1); darker red indicates a stronger LD.

**Figure 4:**
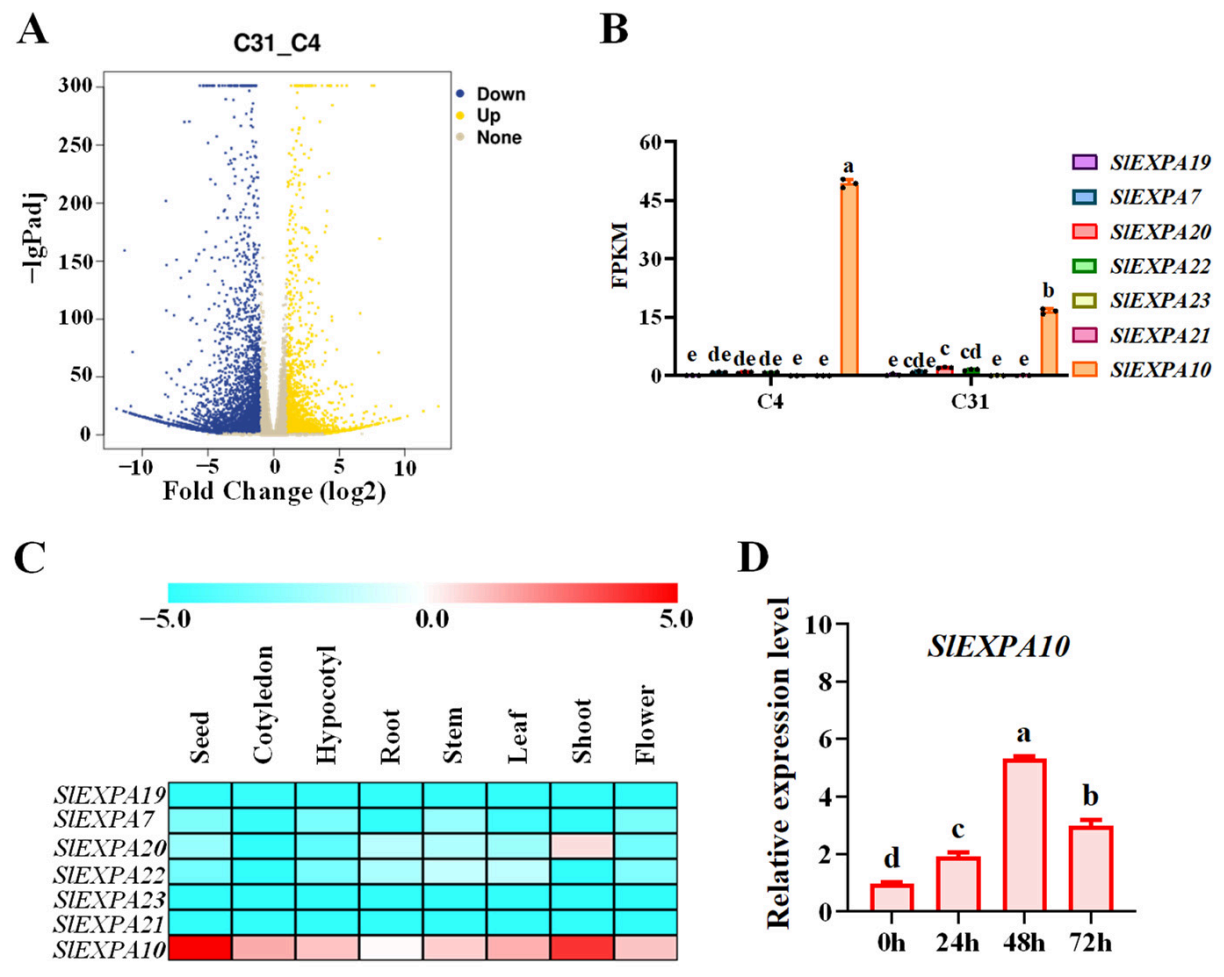
Expression level analysis of *EXPA* genes. (**A**) The volcano plot of differentially expressed genes between C31 and C4 at 48 h after seed imbibition. Blue dots represent down-regulated genes, yellow dots represent up-regulated genes, and gray dots represent genes with no significant differences. (**B**) The expression patterns of seven *EXPA* genes in C4 and C31 at 48 h after seed imbibition. (**C**) The expression patterns of seven *EXPA* genes in different tomato tissues. The expression level data was transformed using log_2_ algorithm and the heatmap shows the color transition from blue (low expression level) to red (high expression level). (**D**) The gene expression level of *SlEXPA10* at 0 h, 24 h, 48 h and 72 h after seed imbibition. Small letters represent significant differences (*p* < 0.05) calculated using one-way ANOVA.

**Figure 5:**
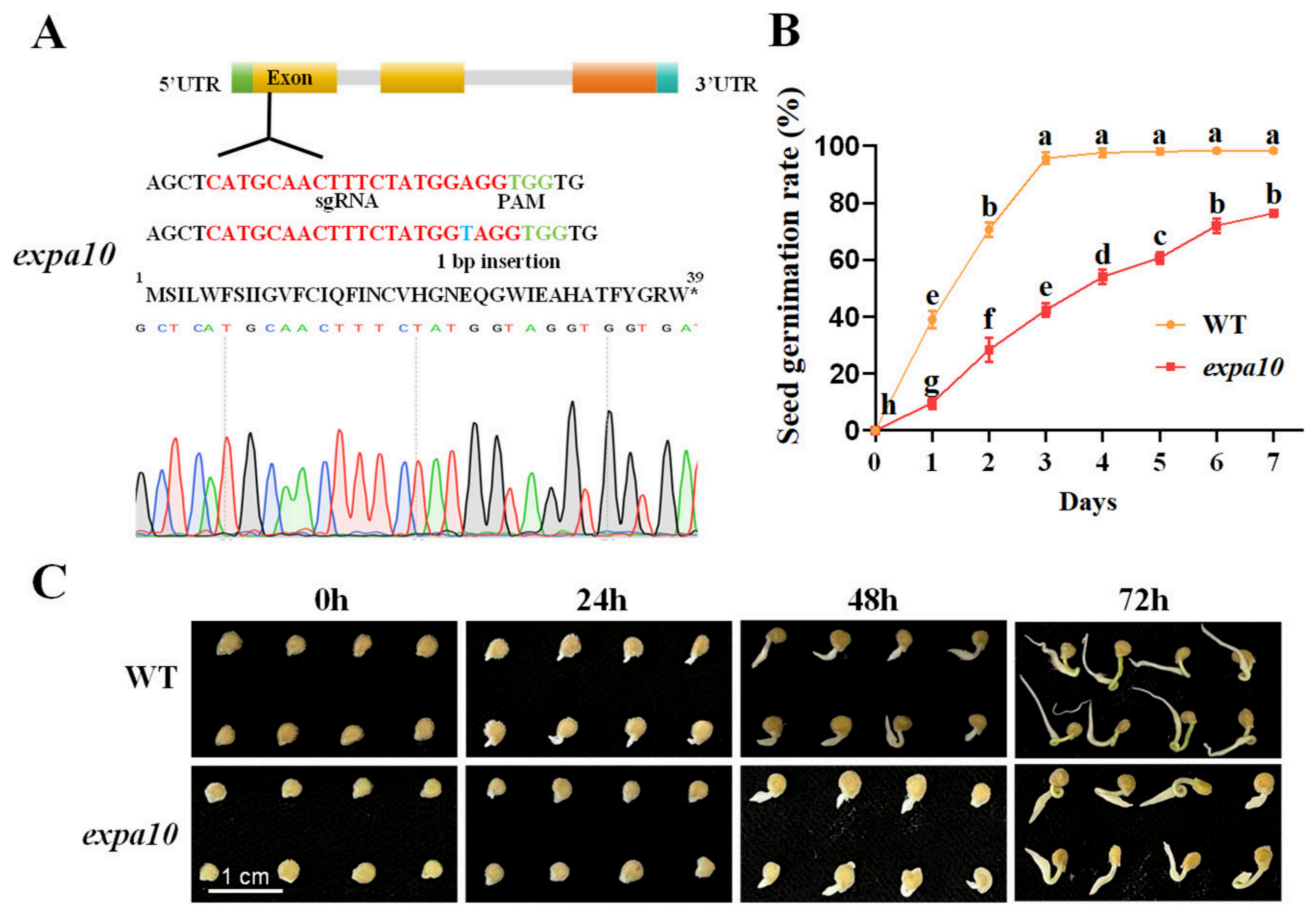
*SlEXPA10* positively regulates seed germination. (**A**) The mutant site of *SlEXPA10*. One base pair insertion in the first exon of *SlEXPA10*, with translation terminated after 39 amino acids. The asterisk represents termination of translation. (**B**) The seed germination rate of wild type (WT) and *expa10* mutant plants for 7 days. Three replicates were conducted and each replicate had 100 seeds. Small letters represent significant differences (*p* < 0.05) calculated using two-way ANOVA. (**C**) Seed germination phenotype of wild type (WT) and *expa10* mutant at 0 h, 24 h, 48 h and 72 h after seed imbibition. Scale bar = 1 cm.

**Figure 6:**
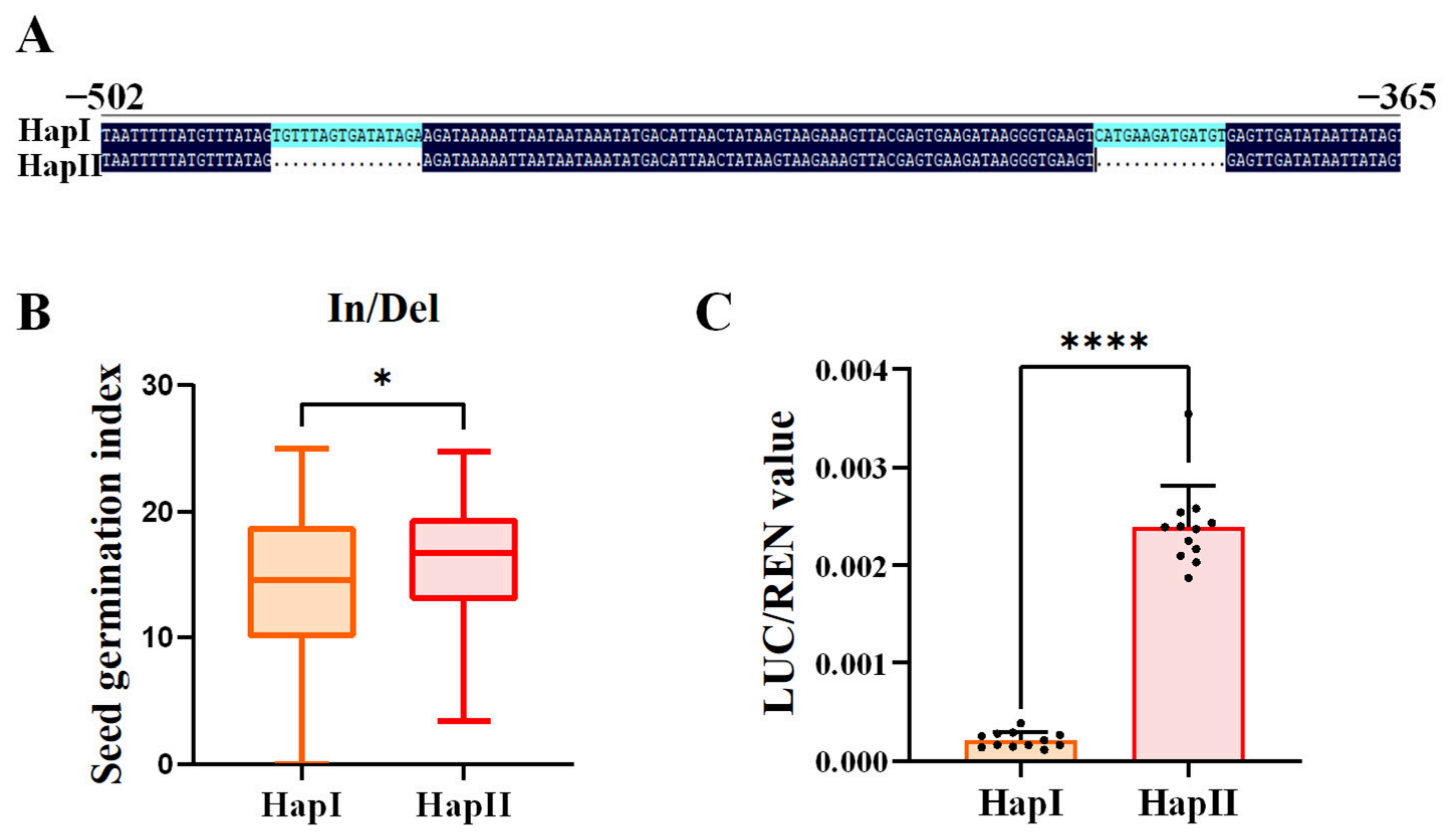
The analysis of InDel variation in the *SlEXPA10* promoter. (**A**) The two haplotypes of InDels in the *SlEXPA10* promoter. (**B**) The seed germination index of the two haplotypes. (**C**) The LUC/REN value of the two haplotype promoters. Significant differences were calculated using one-way ANOVA (* *p* < 0.05, **** *p* < 0.0001).

**Table 1:** Details information of the GWAS loci associated with the seed germination index.

SNP	Chromosome	Position	−log_10_(*P*) Value	REF/ALT
SNP_10222208	1	10222208	8.94	A/G
SNP_59508294	3	59508294	11.11	T/G
SNP_58461279	4	58461279	12.41	AT/A
SNP_50270468	10	50270468	11.15	C/T
SNP_65092668	12	65092668	13.61	A/G

## Data Availability

The raw data of genome resequencing and transcriptome is uploaded to National Genomics Data Center (NGDC), and the accession number is CRA044783 and CRA045345, respectively.

## Supplementary Materials

The following supporting information can be downloaded at https://www.mdpi.com/article/10.3390/ijms27219478/s1.
