# Supplementary material for: Genome-Wide Association Study Identifies Loci and a Candidate Gene Regulating Tomato Seed Germination
Source: Int J Mol Sci. 2026 Oct 23;27(21):9478. doi: 10.3390/ijms27219478 (PMC13763307; doi:10.3390/ijms27219478)
Supplement: Supplementary file 1 [file ijms-27-09478-s001.zip › Figure S1-S4.pdf]

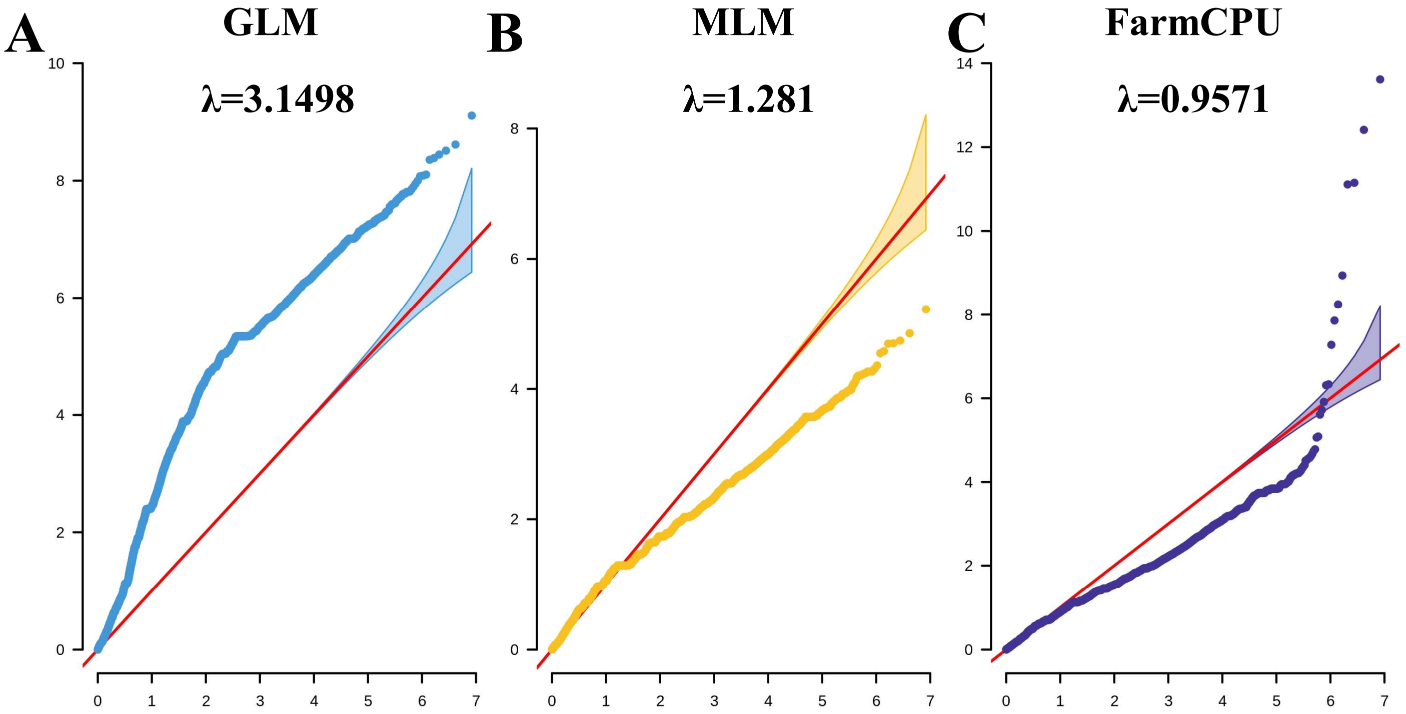

Figure S1. The QQ plot of GWAS analysis. **(A)** Quantile-Quantile (QQ) plot of General Linear Model. **(B)** Quantile-Quantile (QQ) plot of Mixed Linear Model. **(C)** Quantile-Quantile (QQ) plot of Fixed and random model Circulating Probability Unification.

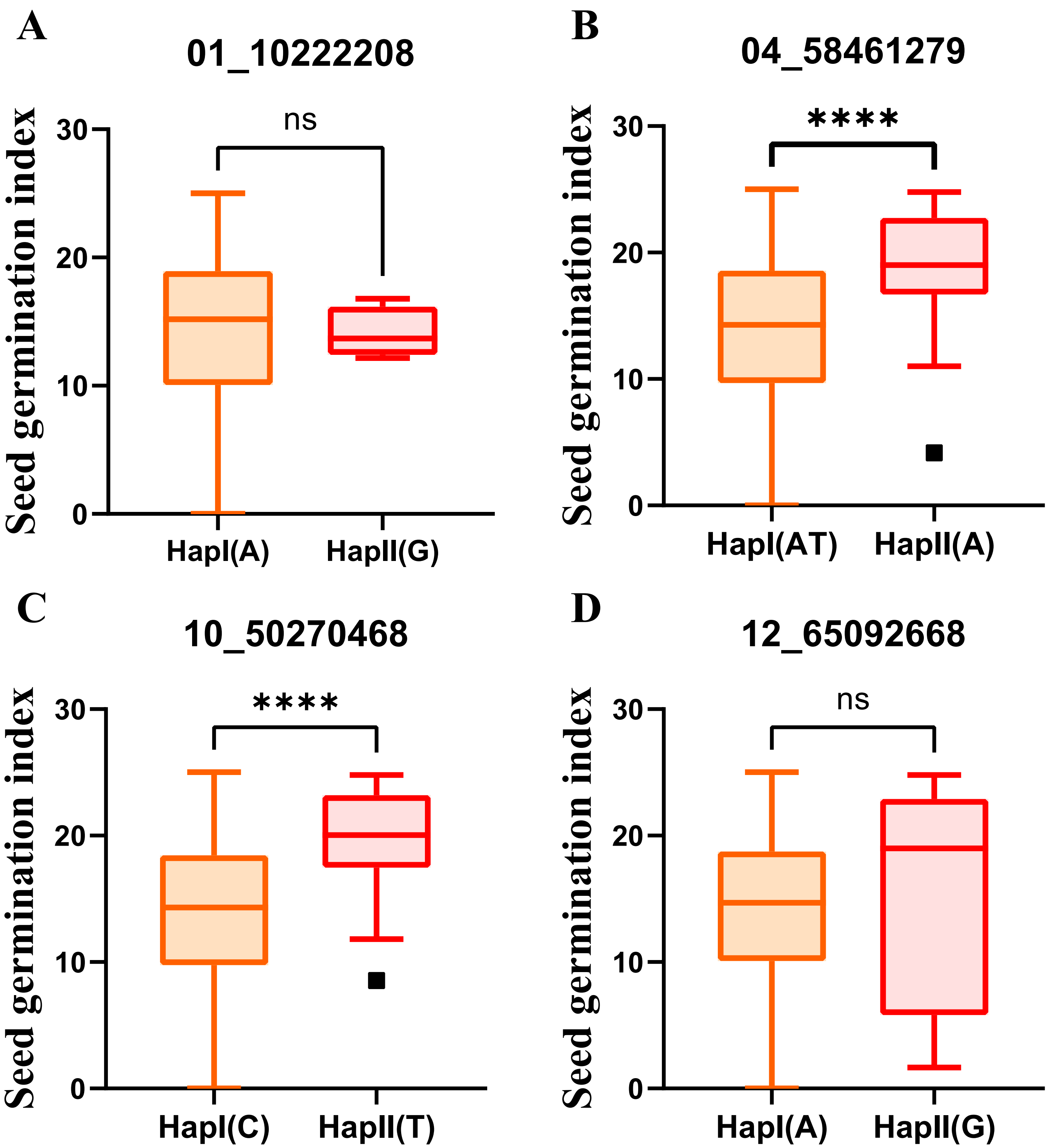

Figure S2. The seed germination index analysis of significant loci. **(A-D)** The seed germination index of two haplotypes of 01_10222208, 04_58461279, 10_50270468 and 12_65092668, respectively. Significant difference was calculated by one-way ANOVA (*****P* < 0.0001). ns means no significant difference.

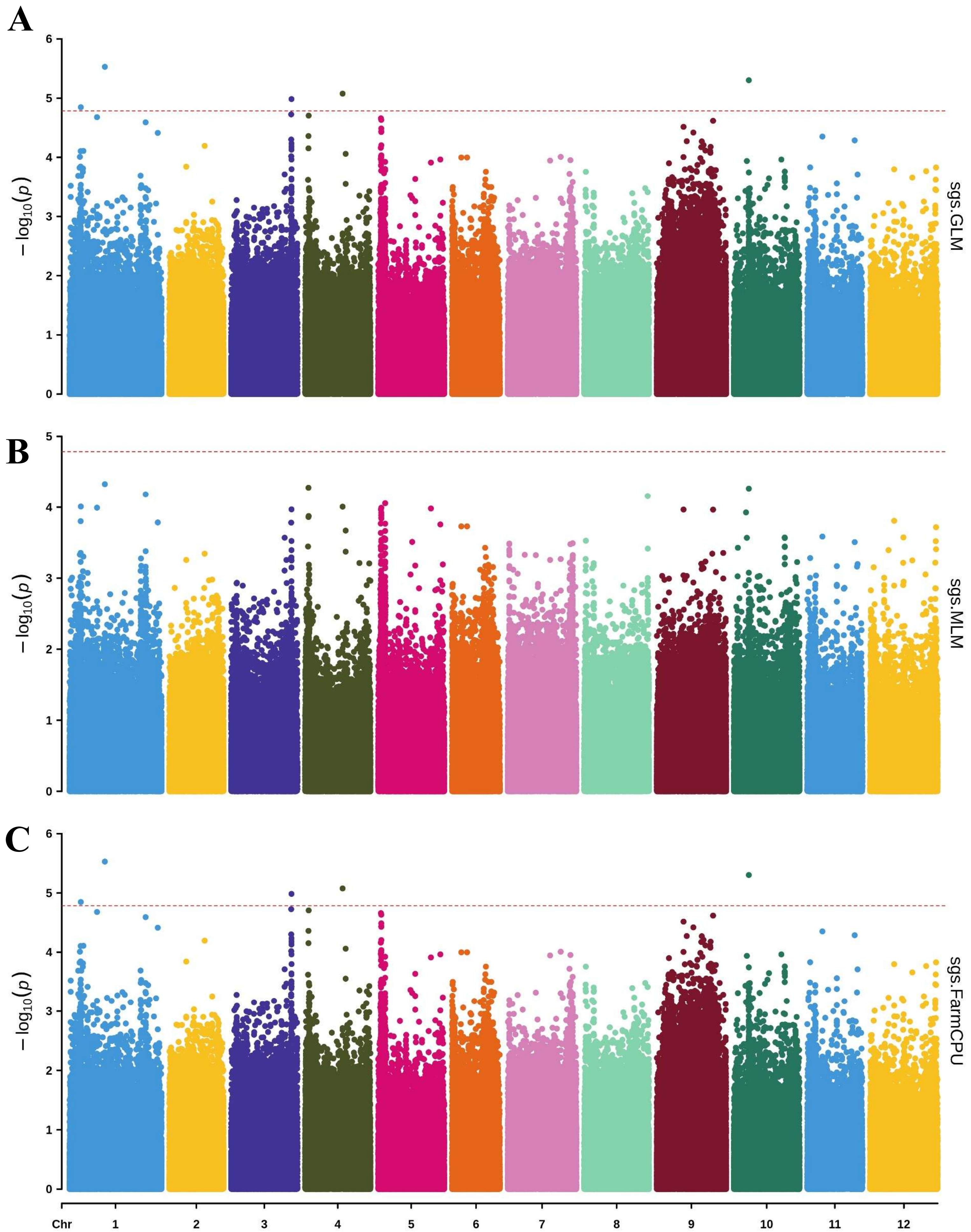

Figure S3. The GWAS analysis with principal component covariates. **(A)** GWAS analysis of General Linear Model (GLM). (B) GWAS analysis of Mixed Linear Model (MLM). (C) GWAS analysis of Fixed and random model Circulating Probability Unification (FarmCPU) model. The dashed horizontal line represents the threshold of GWAS analysis ($-\log_{10}(P) = 4.88$).

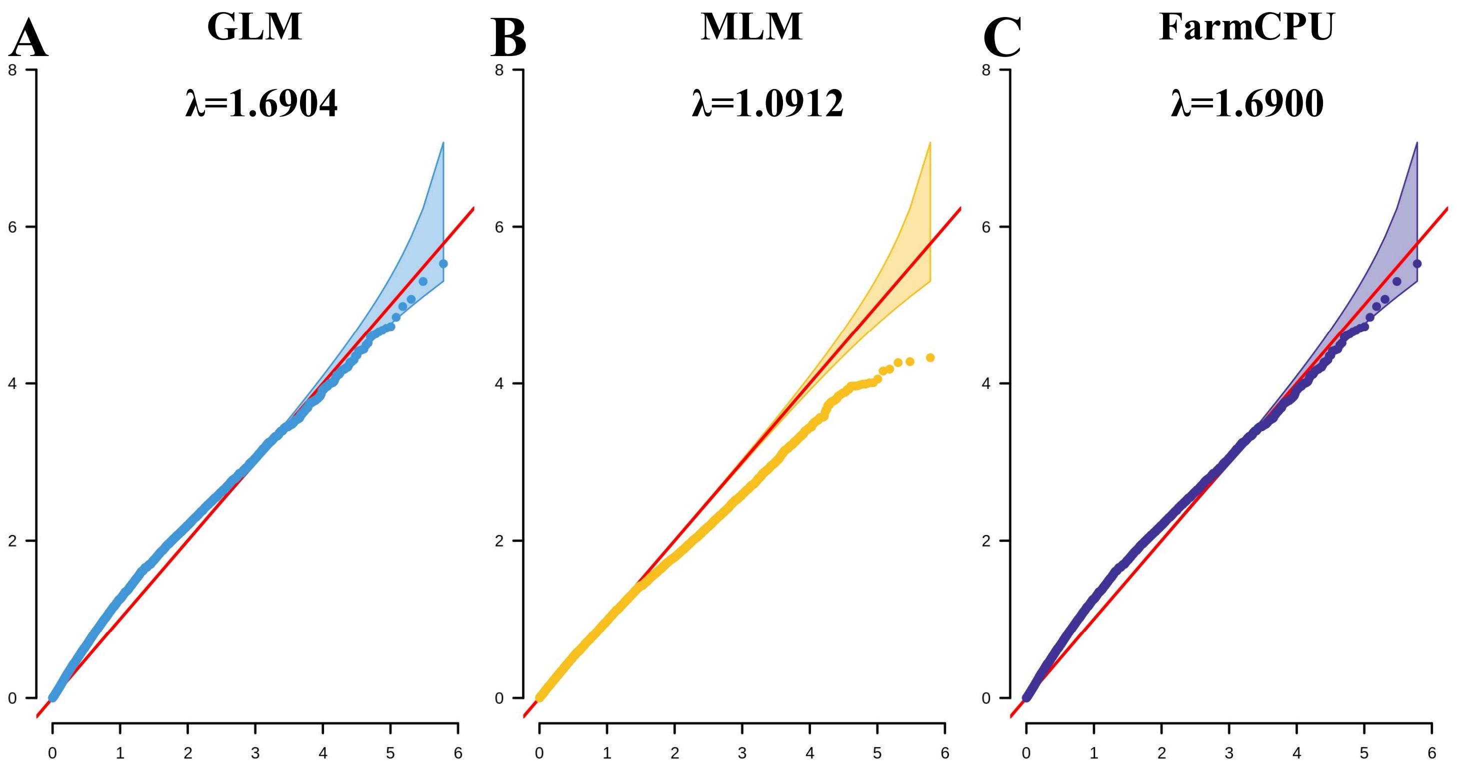

Figure S4. The QQ plot of GWAS analysis with principal component covariates. **(A)** Quantile-Quantile (QQ) plot of General Linear Model. **(B)** Quantile-Quantile (QQ) plot of Mixed Linear Model. **(C)** Quantile-Quantile (QQ) plot of Fixed and random model Circulating Probability Unification. The GWAS analysis incorporating principal components was performed as follows: A genomic kinship matrix was calculated using rMVP (fileKin argument in MVP.Data(fileKin=TRUE) function) and incorporated into (K argument in MVP(K=Kinship) function) to account for genetic relatedness. First 10 columns of principal components were calculated and included as covariates in the GLM, MLM and FarmCPU analyses using rMVP (nPC argument in MVP(nPC=10) function). For FarmCPU, confounding due to population structure and relatedness was addressed through its iterative fixed-effect and random-effect modeling framework with pseu-do-QTN (Quantitative Trait Nucleotide) selection. The remaining parameters were set according to the rMVP default settings. After filtering and linkage disequilibrium screening, a total of 382483 SNPs were retained, and the significance threshold was calculated based on this SNP number.
